# Combined Detoxification and In-situ Product Removal by a Single Resin During Lignocellulosic Butanol Production

**DOI:** 10.1038/srep30533

**Published:** 2016-07-27

**Authors:** Kai Gao, Lars Rehmann

**Affiliations:** 1 Department of Chemical & Biochemical Engineering, The University of Western Ontario, 1151 Richmond St., London, Ontario N6A 3K7, Canada; 2 Department of Biochemical Engineering, AVT – Aachener Verfahrenstechnik, RWTH Aachen University, Worringer Weg 1, 52074 Aachen, Germany

## Abstract

*Phragmites australis* (an invasive plant in North America) was used as feedstock for ABE (acetone-butanol-ethanol) fermentation by *Clostridium saccharobutylicum*. Sulphuric acid pretreated phragmites hydrolysate (SAEH) without detoxification inhibited butanol production (0.73 g/L butanol from 30 g/L sugars). The treatment of SAEH with resin L-493 prior the fermentation resulted in no inhibitory effects and an ABE titer of 14.44 g/L, including 5.49 g/L butanol was obtained, corresponding to an ABE yield and productivity of 0.49 g/g and 0.60 g/L/h, respectively. Dual functionality of the resin was realized by also using it as an *in-situ* product removal agent. Integrating *in-situ* product removal allowed for the use of high substrate concentrations without the typical product inhibition. Resin-detoxified SAEH was supplemented with neat glucose and an effective ABE titer of 33 g/L (including 13.7 g/L acetone, 16.4 g/L butanol and 1.9 g/L ethanol) was achieved with resin-based *in-situ* product removal, corresponding to an ABE yield and productivity of 0.41 g/g and 0.69 g/L/h, respectively. Both detoxification of the substrate and the products was achieved by the same resin, which was added prior the fermentation. Integrating hydrolysate detoxification and *in-situ* butanol removal in a batch process through single resin can potentially simplify cellulosic butanol production.

Butanol, a major product of the acetone-butanol-ethanol (ABE) fermentation, is an important bulk chemical with applications in the productions of solvents, cosmetics and pharmaceuticals. It has also been considered as a renewable liquid transportation fuel with advantages over ethanol^1^,^2^. Traditionally, agriculturally produced substrates such as corn^3^, molasses^4^, and whey permeate^5^ were used for industrial production of ABE through fermentation processes. However, ABE fermentation based on these conventional substrates suffered from increasing prices of feedstock particularly after the second world war and was considered economically unfavourable compared to synthetic routes using petrochemical feedstock^1^. To reduce the cost of fermentative butanol production, alternative low cost substrates such as Jerusalem artichokes^6^,^7^, lignocellulosic materials such as corn cobs, corn stover, switchgrass and phragmites^8^,^9^,^10^,^11^ have been investigated as potential substrates for ABE fermentation. The costs of abundant lignocellulosic biomass tends to be low^12^, however extra process steps such as pretreatment and hydrolysis are required prior to fermentation^13^. Additionally, fermentation inhibitors are often generated during the pretreatment process which either halt or slow down reaction rates of the fermentation^14^. Thus an extra detoxification step is often required to achieve successful fermentation.

Another major problem associated with ABE fermentation is end-product inhibition. For example, butanol titers in a batch fermentation by *C. acetobutylicum* ATCC 824 (most commonly studied butanol producing strain) rarely exceed 13 g/L^1^. Such dilute product streams will cause high energy costs during downstream processing^15^. In fact, economic analyses have suggested that if the butanol titers were raised from 12 g/L to 19 g/L, the separation costs would be cut in half^16^. One of the approaches to avoid inhibitory concentrations is to remove butanol from the cultures while it is being produced and one of the many *in-situ* product removal techniques is through adsorption by polymeric resin. Compared to other types of *in-situ* butanol recovery techniques, adsorption shows superior properties in the stability of extraction phase, biocompatibility, phase immiscibility, reusability, and overall energy efficiency^17^,^18^,^19^,^20^. Dowex^®^Optipore L-493 (poly styrene-*co*-DVB derived resin) has been identified to have high butanol affinity and partitioning coefficient from a pool of commercial resins available^21^ and was recently used in expanded bed adsorption for a fed-batch ABE fermentation from pure glucose^15^. On the other hand, L-493 has been reported to be able to remove various organic compounds from water including phenol^22^ and endocrine disrupting compounds (EDCs)^23^. However, to the author’s best knowledge, this resin has not been used for detoxification of lignocellulosic hydrolysate so far.

A potential lignocellulosic feedstock is *Phragmites australis*, also known as common reed. It is regarded as an invasive species in North America^24^ and has recently been considered as a promising bioenergy crop due to many attractive features such as high biomass productivity (C4 photosynthesis), low requirement on irrigation and fertilizer, easy large-scale planting, and less drying costs due to winter standing^25^. Alkali-pretreated phragmites hydrolysate was successfully fermented to butanol^9^, however, ABE fermentation from acid pretreated phragmites has not yet been reported.

The objectives of the present study are therefore three-fold. The overall goal is the utilization of phragmites with dilute sulphuric acid pretreatment as substrate for ABE fermentation, where detoxification of the feedstock (removal of phenolix, furans, etc.) and detoxification of the fermentation broth (*in-situ* product removal of butanol) are realized with a single sorption resin.

## Results and Discussion

**Figure 1:**
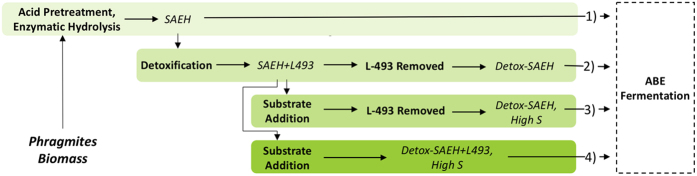
Schematic of the experimental procedures. Stream 1 represent the direct fermentation of the hydrolysate, Stream 2 the fermentation after detoxification, Stream 3 the fermentation with high initial substrate concentration and Stream 4 the fermentation with high substrate and *in-situ* product removal.

The experimental design is schematically illustrated in Fig. 1. *Phragmites australis* was treated with dilute sulphuric acid to generate sulfuric acid pretreated hydrolysates (SAH), which was further hydrolyzed enzymatically (CellicCTec 2, Novozyme, Denmark) to obtain fermentable sugars (SAEH). The effect of inhibitor removal was investigated by fermenting the SAEH directly (stream 1) and after equilibrating with resin L-493 (Dow Canada, Calgary, AB), which was removed prior to fermentation (stream 2). The combined effect of inhibitor removal and *in-situ* product removal was investigated similarly, however the initial glucose concentration was adjusted to 80 g/L (resulting in inhibitory butanol concentrations) and the resin was left in the fermentation vessel (stream 4).

### Dilute sulfuric acid pretreatment and enzymatic hydrolysis

Sulfuric acid pretreatment of the biomass resulted in the typical formation of HMF (Hydroxymethylfurfural) and furfural, as shown in Table 1. The table also shows the sugar profiles before and after enzymatic hydrolysis. It can be seen that during the dilute sulfuric acid pretreatments, considerable amounts of xylose (~16 g/L) were released whereas glucose formation (1.75 to 2.60 g/L) was low, indicating that most of the cellulose was not hydrolysed. As the concentrations of sulphuric acid increased from 0.5% to 2%, HMF levels in the SAH also rose from 0.09 g/L to 0.36 g/L, and the relative content of total phenolic compounds showed significant increase as well; interestingly, furfural concentrations remained stable between 0.09 and 0.12 g/L.

All SAH was subjected to enzymatic hydrolysis in order to obtain fermentable sugars. As shown in Table 1, glucose levels in SAEH increased from 1.75–2.60 g/L to 15.67–17.42 g/L; however, the xylose concentrations showed no significant change after enzymatic hydrolysis. The sugar yield were between 296~317 g sugars per 1 kg of raw phragmites, compared to 385 g of sugars from phragmites pretreated with 1% NaOH as reported previously^9^. The lower sugar yield from acid pretreated phragmites is likely a result of multiple reasons. Phragmites have a relatively high lignin content (~29%)^9^, and lignin is considered a major barrier that prevent cellulolytic enzymes from binding to their cellulose targets^26^. In this regard, alkali pretreatment was shown to be effective in lignin removal, thus resulting in more efficient enzymatic hydrolysis, whereas acid pretreatment has no significant effect on lignin removal, hence a lower sugar yield is expected^27^. In the present study, for the purpose of testing the resin’s function as a detoxification agents, acid pretreated phragmites was directly subjected to enzymatic hydrolysis after neutralization without including an extra washing step to remove degradation products derived from lignocellulose, which may have reduced the efficiency of hydrolysis by enzyme deactivation and precipitation^28^.

The results in Table 1 suggest that increasing the concentrations of sulphuric acid from 0.5% to 2% does not necessarily resulted in higher sugar production in both pretreatment stage and the following enzymatic hydrolysis, but generated higher levels of inhibitors such as HMF and phenolic compounds. Therefore, phragmites hydrolysates pretreated with 0.5% (v/v) H_2_SO_4_ was used for further studies (lower acid concentrations resulted lower sugar production, data not shown).

### Detoxficiation and fermentation

**Figure 2:**
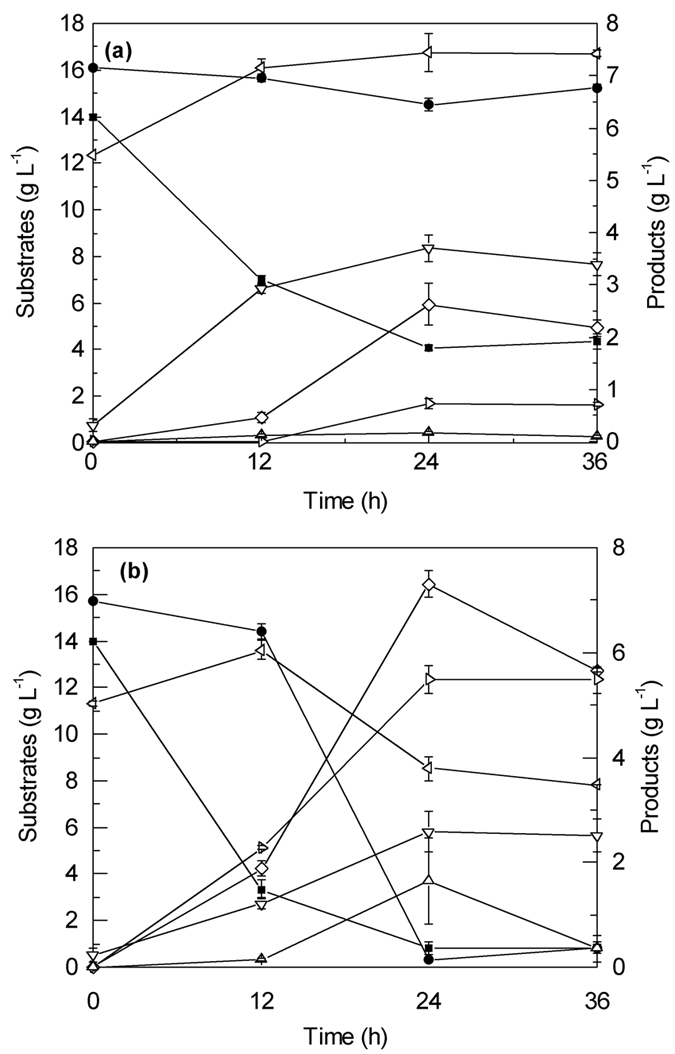
Profiles of ABE Fermentation from undetoxified (**a**) and detoxified (**b**) phragmites hydrolysate at 37 °C, 150 rpm (-■-, glucose; -●-, xylose; -◁-, acetic acid; -▽-, butyric acid; -◊-. acetone; -▷-, butanol; -△-, ethanol).

The ability of *C. saccharobutylicum* to ferment the hydrolsate to butanol was evaluated with and without the detoxification step. Time profiles of the fermentation of undetoxified SAEH are shown in Fig. 2a. It can be seen that *C. saccharobutylicum* was able to utilize the glucose present in the undetoxified hydrolysate. Initially, 13.96 g/L glucose and 16.12 g/L xylose were present in the hydrolysate. After 24 h, 9.88 g/L glucose was consumed, corresponding to 71% of the initial glucose concentration. However, xylose utilization by the bacteria was severely inhibited. The fermentation resulted in a total ABE production of 3.53 g/L, including 2.63 g/L acetone, 0.17 g/L ethanol, and 0.73 g/L butanol, as well as 7.44 g/L acetic acid (most of it a product of the pretreatment) and 3.70 g/L butyric acid. Interestingly, the initial 0.04 g/L HMF and 0.07 g/L furfural present in the undetoxified hydrolysate were not detected after 12 h of fermentation (data not shown), indicating that the strain has the ability to metabolize HMF and furfural, which could possibly explain results from previous studies showing that supplementation of HMF and furfural (0.3 to 2 g/L) can actually help improve ABE fermentation in terms of cell concentration and solvents production^29^. However, the overall sugar utilization and solvent production was low and other toxic compounds present in the untreated hydrolysate likely inhibit the bacteria’s growth and ability to utilize the sugars especially xylose. This is likely caused by phenolic compounds, as it was reported that ferulic acid (a phenolic acid) as low as 0.3 g/L was a strong inhibitor to cell growth and solvent production^29^. Other toxic compounds also include phenol, р-coumaric acids, and syringaldehyde, etc. Therefore, to facilitate better sugar consumption and ABE yield, the hydrolysate likely needs to be detoxified to improve its fermentability.

The effect of detoxification by resin L-493 on phragmites hydrolysate (pretreated with 0.5% H_2_SO_4_) was investigated. As shown in Table 2, the level of glucose, xylose, acetic acid and HMF remained almost unchanged after detoxification with the resin (30 g/L). However, the detoxified hydrolysate showed remarkable decrease in furfural content and total phenolic compounds, a 95% decrease in relative content of total phenolic compounds was observed after detoxification. These results indicated the polymer resin L-493 has a low affinity for the fermentation substrate such as glucose and xylose, but a much higher affinity for the phenolic compounds in the hydrolysate. In comparison, significant sugar reduction (15%) was observed when sulphuric acid treated corn fiber was detoxified with resin XAD-4^30^, rendering L-493 more suitable for this purpose.

Total phenolic compounds were semi-quantitatively estimated^31^ in order to compare pretreatment and detoxification condition. The values presented in Table 1 are an excellent evaluation tool, but not all phenolic species contributing to the ‘total phenolic compounds’ value inhibit cells equally. Although it is difficult to fully characterize the many types of phenolic compounds present in the phragmites hydrolysates, some of the most representative phenolic compounds have been quantified via HPLC (High Performance Liquid Chromatography), and a summary is given Table 3. The profiles of phenolic compounds present in lignocellulose hydrolysates are highly dependent on the types of feedstocks and pretreatment conditions, however the concentrations reported here are comparable with results available elsewhere^32^. As can be seen from the table, a series of phenolic compounds has been generated during dilute H_2_SO_4_ pretreatment. The relative abundance of specific compounds changed with acid concentration, with some compounds such as p-coumaric acid, syringaldehyde, and ferulic acid decreasing in concentration as the acid concentration was increased from 0.5% to 2%, while phenol itself slightly increased. No individual phenolic compounds could be detected with the given methodology after detoxification, indicating that resin L-493 can be effectively used for the removal of phenolic compounds.

The fermentation results with detoxified phragmites hydrolysate are shown in Fig. 2b. Initially 13.98 g/L glucose and 15.72 g/L xylose was present in the hydrolysate. After 24 h of fermentation, the culture was able to utilize 13.17 g/L glucose and 15.37 g/L xylose, leaving 0.81 g/L glucose and 0.35 g/L xylose unutilized. The fermentation resulted in an ABE production of 14.44 g/L, including 7.31 g/L acetone, 1.64 g/L ethanol, and 5.49 g/L butanol, corresponding to an ABE yield and productivity of 0.49 g/g and 0.60 g/L/h, respectively. The productivity and yield were close to that of a selected *C. acetobutylicum* strain after random mutagenesis reported by Jang *et al*.^33^, however Jang *et al*. operated at high initial substrate concentrations, hence achieved a higher product titers. The physiological stress caused by high solvent concentrations typically results in yield reduction, which can potentially be overcome through ISPR techniques such as the resign employed in this study. At 36 h, the culture accumulated 3.48 g/L acetic acid and 2.51 g/L butyric acid. Much less acetic acid was remained from detoxified SAEH compared to 7.44 g/L from original SAEH, which may indicate that toxic compounds in original SAEH might reduce the strain’s ability to assimilate acetic acid. Only trace amounts of HMF and furfural were detected at the beginning of fermentation, and both compounds were not detected after 12 hrs. These results suggested that detoxification by resin L-493 is an efficient way to improve the fermentability of acid hydrolysate of lignocellulosic biomass. The ABE yield was improved from 0.12 g/g without detoxification to 0.49 g/g with detoxification by polymer resin, which compared well to previous reported solvent yields from mixed sugars (glucose and xylose) and corn cobs hydrolysates by the same strain^8^.

### Adsorption and desorption of butanol by resin L-493

**Figure 3:**
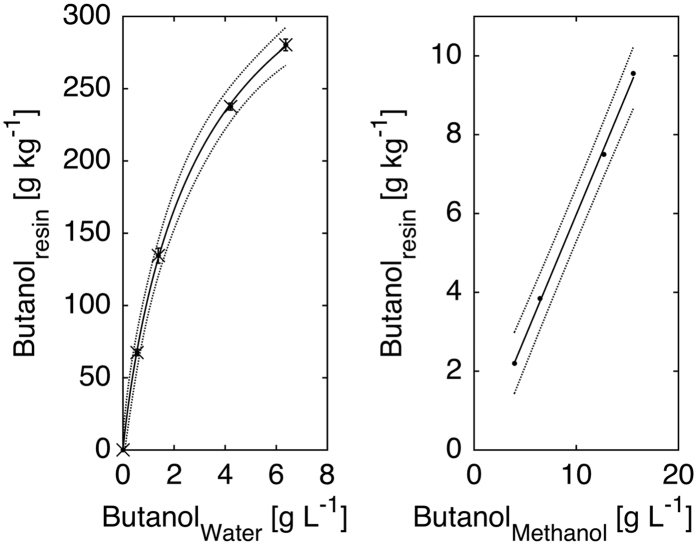
Butanol adsorbed to resin as a function of the equilibrium butanol concentrations in (**a**) aqueous phase (50 g/L resin) and (**b**) methanol phase (333 g/L resin) with various initial butanol concentrations (4, 8, 16 and 20 g/L) at 37 °C after shaking for 12 h (220 rpm). Solid squares represent butanol adsorbed by the resin (g/kg); the solid lines represent the fitted Langmuir isotherm (water) and linear isotherm (methanol) while the dotted lines represent the 95% prediction limits.

The selected resin was capable of removing fermentation inhibiting phenolic compounds from the hydrolysate, as discussed above. To exploit the material to its fullest it was investigated for its ability to additionally function as an *in-situ* product removal agent. The equilibrium distribution of butanol between water and the resin is shown in Fig. 3a. The equilibrium favors sorption to the resin and the data is in good agreement with values reported elsewhere^21^. The isotherm data could be fitted with the Langmuir model and the following relationship was found:


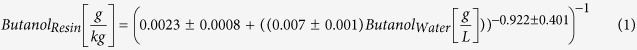


Butanol was re-suspended from the resin via equilibration in methanol. This step was used for analytical purposes to quantify the total amount of butanol produced during fermentation with *in-situ* product removal. An isotherm is shown in Fig. 3b and over the tested range a simple linear partitioning coefficient of K_R/Me _= 0.6214 ± 0.0651 
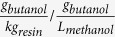
 could be determined for the equilibrium data. The relationship was used to determine the amount of butanol in the resin at the end of the fermentation after desorption of butanol into methanol followed by HPLC analysis. This method was validated by equilibrating target butanol concentrations in fermentation media (16 and 20 g/L) with the resin (50 g/L). The aqueous butanol concentration was effectively reduced to sub-inhibitory levels (Table 4) and removal of the resin from the aqueous phase and submersion in methanol result in high butanol recovery.

### Integration of hydrolysate detoxification and fermentation with *in-situ* butanol removal

**Figure 4:**
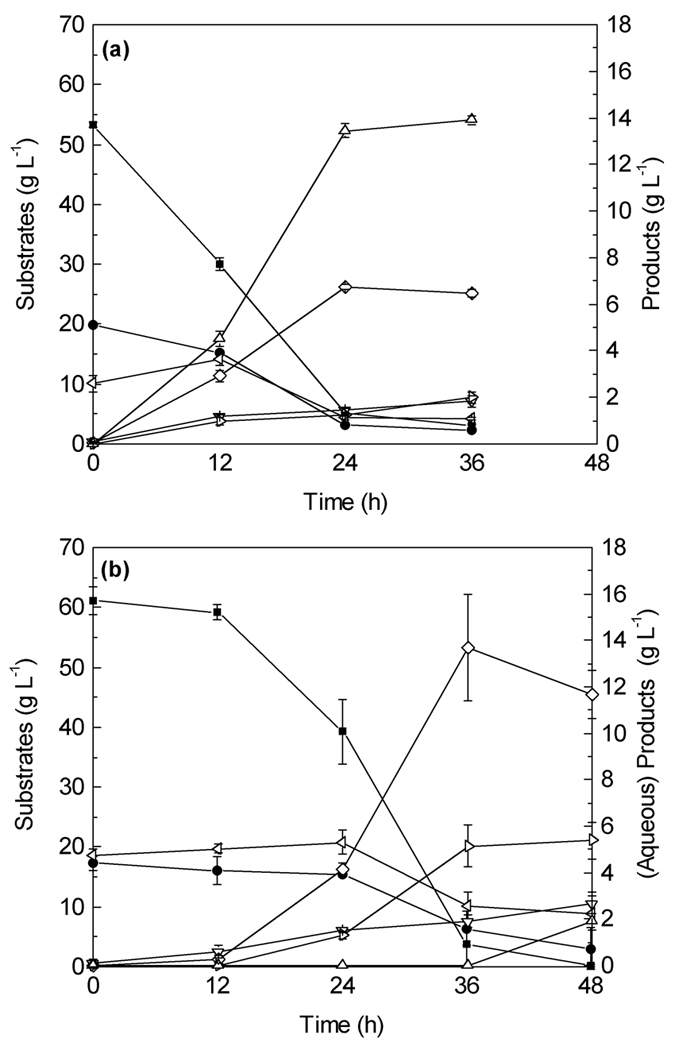
Profiles of ABE Fermentation from (**a**) mixed sugar and (**b**) phragmites hydrolysate (supplemented with glucose) with *in-situ* product removal at 37 °C, 150 rpm (-■-, glucose; -●-, xylose; -◁-, acetic acid; -▽-, butyric acid; -◊;-. acetone; -▷-, butanol; -△-, ethanol).

One of the major drawbacks of the ABE fermentation is low product titers due to the toxicity of butanol to the producing organisms. The tested hydroysis conditions did not yield high enough sugar concentrations to result in product inhibition at the end of the fermentation. Therefore, the hydrolysate was supplemented with pure sugars in order to achieve higher product titers. A control fermentation (Fig. 4a) with mixed sugar (53.31 ± 0.45 g/L glucose and 19.93 ± 0.06 g/L xylose) was performed to compare the results of fermentation with *in-situ* butanol removal (Fig. 4b). During the control fermentation, 48.30 ± 0.28 g/L glucose and 16.78 ± 0.08 g/L xylose were consumed after 24 h of fermentation, leaving behind 8.16 ± 0.76 g/L sugar unutilized. In the end of fermentation, 21.45 ± 0.42 g/L ABE was produced (including 6.73 ± 0.72 g/L acetone, 13.45 ± 0.31 g/L butanol and 1.27 ± 0.05 g/L ethanol), corresponding to an ABE yield and productivity of 0.33 g/g and 0.89 g/L/h, respectively. The results from control fermentation clearly show that the strain is not able to completely utilize all the sugars available in the fermentation broth with an initial sugar concentration of ~70 g/L, likely due to product inhibition^34^.

In order to maintain sub-inhibitory product concentrations an ABE fermentation from SAEH was carried out with *in-situ* butanol removal by resin L-493 (50 g/L resin dosage) (glucose supplemented, Fig. 4b). Before the fermentation started, SAEH was detoxified with the same resin. Initially, ~60 g/L glucose (15 g/L from SAEH and 45 g/L from pure glucose) and 17 g/L xylose (from SAEH) were present in the fermentation media. Slow initial utilization of sugars (both glucose and xylose) was observed within the first 12 h of fermentation, compared to a rapid glucose utilization immediately after inoculation with detoxified SAEH at lower initial sugar concentrations (Fig. 2b). The only difference here is the initial sugar concentration (80 vs 30 g/L), therefore, substrate inhibition in combination with slight toxicity caused by non-removed inhibitors is likely the major reason to cause the delay in sugar utilization, which was not observed with pure sugars. The strain started to use glucose rapidly after 12 h; however, xylose utilization was not obvious until 24 h. Similar mixed sugar (mainly glucose and xylose) usage pattern was reported previously, which is mainly due to the carbon catabolite repression found in many bacteria^8^,^35^,^36^. After 48 h of fermentation, 0.19 g/L glucose and 3.05 g/L xylose was left unutilized in the fermentation media (Table 5).

During the first 24 h, acetic acid was produced by the cultures and a maximum concentration of 5.31 g/L was obtained at 24 h, indicating that the cultures were going through acidogenesis. After 24 hrs, a significant acetic acid re-assimilation was observed as the acetic acid level was reduced to 2.17 g/L after 60 h. In comparison, no significant re-assimilation of butyric acid was observed during the fermentation.

Solvent production was observed after 24 hrs. The butanol concentration in the aqueous phase reached 5.38 g/L after 48 hrs (Table 5), which is well below the butanol threshold level in a batch fermentation (~12–13 g/L)^16^. Additional butanol was sorbed to the resin, up to 263 mg/g can be estimated, assuming equation 1 is adequate for the system also containing additional fermentation products as well as the by-products from the acid hydrolysis. As shown in Fig. 4b, selective adsorption of butanol by resin L-493 makes acetone a predominant product in the aqueous phase and a maximum acetone concentration of 13.70 g/L was achieved at 36 h.

After the fermentation was stopped, desorption experiments with methanol were carried out to recover the butanol that had been absorbed by the resin. Substantial amount of butanol (11.02 g/L), acetone (2.97 g/L and butyric acid (1.92 g/L) were recovered in the methanol phase (30 ml), while only trace amounts of glucose, xylose and ethanol were detected. The total butanol produced (161.4 mg in aqueous phase and 330.6 mg in the resin) is equivalent to an effective butanol titer of 16.4 g/L. The measured value is slightly below what would have been expected based on the aqueous phase concentration and the resulting equilibrium concentration in the resin based on eq. 1. However, the isotherms shown in Fig. 3 were measured for a system containing only water and butanol. In the case of the fermentation acetone and ethanol were also present, and the resin was loaded with the phenolic by-product from the acid hydrolysis, which were adsorbed during the detoxification stage. In the end, an effective ABE production of 33 g/L was obtained, corresponding to an ABE yield and productivity of 0.41 g/g and 0.69 g/L/h, respectively.

The major contribution of the current study is to use Optipore L-493 for both hydrolysate detoxification and *in-situ* butanol removal in a single process. It was recently reported that Optipore L-493 has been considered as effective *in-situ* butanol absorbent and used in expanded bed adsorption for a fed-batch ABE fermentation^15^,^21^. However, this resin has not been used for detoxification of lignocellulosic hydrolysate yet to the authors’ knowledge.

The inhibiting effect of dilute sulfuric acid pretreatment on ABE fermentation has been observed among a wide range of substrates, such as dried distillers’ grains and solubles (DDGS), corn fiber, barley straw, corn stover and switchgrass^30^,^37^,^38^,^39^. Detoxification techniques such as overliming and inhibitor removal by resin XAD-4 have been employed but unsatisfied results were obtained (Table 6), and the cost of chemicals during overliming is also making the process less economic^30^. Results presented in this study shows that a previous contact with resin L-493 alone can efficiently remove inhibitors present in the phragmites hydrolysate. Phragmites used in this study is a typical herbaceous plant and has similar compositions compared to other cellulosic feedstocks studied before^40^; levels of toxic compounds generated in the present study are also comparable to those found in other feedstocks such as wheat straw, barley straw and switchgrass^38^. Therefore, it is reasonable to conclude that resin L-493 has potential for inhibitor removal for other lignocellulosic hydrolysates as well. The application of resin L-493 in both detoxification and *in-situ* product removal fermentation makes it possible to simplify the process for cellulosic butanol production while increasing the process’s production efficiency.

## Methods

### Raw material

Raw phragmites were collected near London, Ontario, Canada. Air-dried biomass (moisture content ~5%) was first cut into small pieces (~5 cm × 2 cm) and then grounded by a grinder (IKA^®^ MF10, Sigma Aldrich) to pass a 2 mm sieve and stored in sealed plastic bags at room temperature until used for pretreatment. *C. saccharobutylicum* DSM 13864 was purchased from Leibniz Institute DSMZ - German Collection of Microorganisms and Cell Cultures. Cultures of this strain were routinely maintained as spore suspensions at 4 °C in seed cultures. Preparation of inoculums has been reported elsewhere^7^.

### Dilute sulfuric acid pretreatment and enzymatic hydrolysis

Biomass (20 grams of phragmites) was mixed well with 180 ml 0.5%, 1% and 2% (v/v) sulphuric acid in 500 ml screw-capped bottles, and soaked for 15 min before thermal treatment for 60 min at 121 °C (AMSCO Eagle Series 2041 Autocalve, Steris, Mentor, OH). After the pretreatment, the slurries (SAH) were cooled to room temperature, and samples were taken to measure the sugar concentration and inhibitor levels in the hydrolysate.

Before enzymatic hydrolysis, the pH of the SAH was adjusted to 5 by adding 10 M NaOH. Cellic CTec2 (kindly donated by Novozyme) was dosed to give a filter paper activity of 15 FPU/g biomass. Hydrolysis was performed in an orbital incubator (InforsMulitron, Infors Switzerland) at 50 °C, 150 rpm for 72 h. Samples were periodically taken for sugar analysis. The enzymatic hydrolysates of dilute sulfuric acid pretreated phragmites (SAEH) was transferred to 50 ml falcon tubes and centrifuged at 3,500 rpm (ST40R, Thermo Scientific) to remove sediments. The supernatant was stored in pre-sterilised flasks at 4 °C for detoxification and fermentation studies.

### Detoxification and fermentation

Dowex^®^ Optipore L-493 (a commercial polymer resin, chemical name poly (styrene-*co*-DVB)) was used for detoxification studies. The resin was dried at 50 °C in a convection oven for 24 h before use. Approximately 0.9 g of resin was added to 50 ml serum bottles, then 30 ml of the SAEH (supernatant collected from previous step) was transferred to the same bottle, corresponding to a resin concentration of 30 g/L. The bottle was capped air tight and placed in an incubator (InforsMultitron, Infors Switzerland) at 37 °C, shaking (200 rpm) for 12 hours to allow detoxification. Control experiments were carried out without the addition of polymer resin as a comparison. Samples were taken at the end of experiments to measure the concentration of sugars, acids, furfural, HMF and total phenolic compounds.

About 25 ml of detoxified SAEH (without resin) was transferred to a pre-sterilized 125 ml Erlenmeyer flask by aspiration through syringe. Nutrients for bacteria growth were added as reported previously^8^; the media contained (per litre) 3 g yeast extract, 5 g peptone, 2 g ammonium acetate, 2 g NaCl, 3 g MgSO_4_, 1 g KH_2_PO_4_, 1 g K_2_HPO_4_, and 0.1 g FeSO_4_. No extra carbon source was supplemented. The pH of the media was adjusted to around 6.5 by 1 M NaOH/H_2_SO_4_ before fermentation started. The flasks containing the media were heat shocked in a water bath (80 °C) for 10 min to remove dissolved oxygen and transferred to an anaerobic chamber(Model 855-ACB, Plas Labs, Lansing, MI) for anaerobiosis. *Clostridium* cells cultured at a temperature of 37 °C for 10–12 h without any agitation were used as the inoculum for fermentation studies at an inoculum size of 10% (v/v). Samples were taken every 12 h until fermentation stopped. Fermentation with undetoxified hydrolysate was also carried out as control experiments.

### Fermentation with *in-situ* butanol removal

Supernatant of SAEH (30 ml) was transferred to pre-sterilised shaking flasks (125 ml). After nutrients were added, pure glucose was supplemented to adjust the total sugar concentration in fermentation media to 80 g/L. 1.5 g polymer resin was added to the media before the pH was adjusted to 6.5. The flasks were heat shocked and transferred to anaerobic chamber to equilibrate overnight (37 °C, 200 rpm). The fermentation media containing polymer resins was inoculated with 3 ml seed cultures to initiate fermentation. Samples were taken every 12 h for measurements of sugars, acids, and solvents.

### Equilibrium adsorption isotherm and desorption

Butanol adsorption experiments were performed using 30 ml serum bottles containing 0.5 g L-493 resin (oven dried) and 10 mL aqueous butanol solution with concentrations of 4 g/L, 8 g/L, 16 g/L and 20 g/L. Butanol equilibrium data between methanol and resin were generated similarly (1 gram of resin was immersed in 3 ml methanol containing same concentrations of butanol as mentioned above). The bottles were capped tightly and agitated for 12 h (37 °C, 220 rpm). At the end of the adsorption experiments, samples were taken by aspiration through a syringe to measure the butanol concentration in the liquid phase. The amount of butanol adsorbed by the resin (mg/g) was calculated via the mass balance as reported elsewhere^41^.

Desorption of butanol from equilibrated resins (both aqueous phase and fermentation with *in-situ* product removal) was performed by first removing free bulk liquid (fermentation media) by syringe aspiration. Then methanol (20 ml pure methanol/g oven-dried resin) was injected. The bottle was sealed properly and placed into an air-bath incubator (InforsMultitron, Infors Switzerland) at 37 °C (220 rpm) for 12 h. Samples were taken to measure the concentrations of butanol and other components desorbed from the resin via HPLC.

### Analytical methods

Concentrations of glucose, xylose, acetic acid, butyric acid, and ABE were determined by HPLC using an Agilent 1260 liquid chromatography system (Agilent Technologies, Inc., CA, USA) equipped with a Hi-plex H column (7.7 × 300 mm) at 15 °C and a refractive index detector. 5 mM H_2_SO_4_ was used as mobile phase with a flow rate of 0.5 ml/min. Furfural and HMF were measured by a diode array detector (DAD) (G1315C, Agilent) with a wavelength of 280 nm. An Agilent Poroshell 120 EC-C18 column (4.6 × 100 mm) was used with the same HPLC system to measure individual phenolic compounds. The signal of DAD was set to 270 nm and a gradient method was used. Briefly, the gradient started and held at 95% mobile phase A (0.1% formic acid) and 5% mobile phase B (acetonitrile); then ramped to 40% B, and then re-equilibrated to the initial condition. The flow rate was kept at 1 ml/min. All samples collected were centrifuged at 12,000 rpm for 10 min and filtered through 0.2 μm filters before analysis.

Total phenolic compounds were semi-quantitatively estimated based on a slightly modified method reported elsewhere (Li *et al*.^31^). In brief, the hydrolysate was diluted with distilled water and the absorbance was determined at 280 nm by an UV-Visible spectrophotometer (Evolution 60S, Thermo Scientific) with distilled water as reference. The OD value is presented in this paper as a relative measure of the total phenolic compounds in solution.

ABE productivity (g/L/h) was calculated as the maximum ABE concentration achieved (g/L) divided by the fermentation time (h). ABE yield was calculated as the maximum amounts of solvents (ABE) produced divided by the amount of sugar available initially in the fermentation media and are expressed as g/g.

Triplicates were carried out for all experiments and the average numbers and standard errors are shown in tables and figures.

## Additional Information

**How to cite this article**: Gao, K. and Rehmann, L. Combined Detoxification and In-situ Product Removal by a Single Resin During Lignocellulosic Butanol Production. *Sci. Rep.* **6**, 30533; doi: 10.1038/srep30533 (2016).

## Figures and Tables

**Table 1:** Profiles of sugars and inhibitors after pretreatment at different acid concentrations (SAH) and subsequent enzymatic hydrolysis (SAEH).

Substrate	H_2_SO_4_conc. % (v/v)	Glucose (g/L)	Xylose (g/L)	Acetic acid (g/L)	HMF (g/L)	Furfural (g/L)	Total phenolic compound (OD)
SAH	0.5	1.75 (0.04)	16.03 (0.27)	4.53 (0.05)	0.09 (0.01)	0.10 (0.00)	92(2)
	1.0	2.20 (0.03)	16.61 (0.05)	4.82 (0.02)	0.20 (0.00)	0.12 (0.00)	144(6)
	2.0	2.60 (0.06)	16.40 (0.55)	5.32 (0.56)	0.36 (0.06)	0.09 (0.01)	263(4)
SAEH	0.5	15.67 (0.62)	17.22 (0.28)	5.33 (0.49)	0.09 (0.01)	0.08 (0.00)	85(3)
	1.0	17.42 (0.74)	17.75 (0.09)	4.83 (0.08)	0.18 (0.01)	0.11 (0.01)	135(5)
	2.0	16.81 (1.10)	16.82 (0.05)	4.85 (0.00)	0.28 (0.02)	0.09 (0.00)	260(4)

Average values of triplicate experiments with standard error in brackets.

**Table 2:** Detoxification of SAEH pretreated with 0.5% H_2_SO_4_ by L-493.

	Glucose (g/L)	Xylose (g/L)	Acetic acid (g/L)	HMF (g/L)	Furfural (g/L)	Total phenolic compounds (OD)
Undetoxified	15.46 ± 0.09	17.93 ± 0.06	4.08 ± 0.00	0.06 ± 0.01	0.09 ± 0.00	82 ± 4
Detoxified	15.00 ± 0.17	17.06 ± 0.08	3.76 ± 0.06	0.04 ± 0.02	0.02 ± 0.01	4 ± 0

**Table 3:** Concentrations of selected phenolic compounds present in the acid hydrolysates of phragmites (before and after resin detoxification).

Compounds (mg/L)	Undetoxified			Detoxified		
	0.5% H_2_SO_4_	1% H_2_SO_4_	2% H_2_SO_4_	0.5% H_2_SO_4_	1% H_2_SO_4_	2% H_2_SO_4_
4-Hydroxybenzoic acid	3.8	5.7	5.3	—	—	—
Phenol	35.1	38.2	42.4	—	—	—
p-Coumaric acid	79.1	31.2	10.8	—	—	—
Syringaldehyde	9.7	6.4	5.5	—	—	—
Ferulic acid	70.5	36.4	13.4	—	—	—
2,6-Dimethoxyphenol	18.1	15.0	20.2	—	—	—

–undetected; values presented are average of triplicate experiments.

**Table 4:** Sorption and desorption of butanol by resin L-493.

Initial [Butanol]_Aq_(g/L)	Equilibrated [Butanol]_Aq_(g/L)	[Butanol]_methanol_(g/L)	Butanol Recovery %
16	4.21	11.14	96
20	6.37	13.61	99

**Table 5:** Profiles of fermentation of detoxified SAEH with *In-situ* butanol removal (48 h).

Glucose (g/L)	Xylose (g/L)	Acetic acid (g/L)	Butyric acid (g/L)	Acetone (g/L)	Ethanol (g/L)	Butanol (aq) (g/L)	Effective Butanol (g/L)
0.19 ± 0.26	3.05 ± 3.47	2.25 ± 0.73	2.7 ± 0.46	11.66 ± 1.05	1.93 ± 0.00	5.38 ± 0.80	16.4*

*The effective butanol concentration was estimated as the total amount of butanol in the system (aqueous and in resin) divided by the total volume of aqueous phase.

**Table 6:** Performance of different types of resin used as inhibitor and butanol absorbents.

Substrate	Resin	Detoxification/*In-situ* butanol removal	Performance			Ref.
			Effective butanol (ABE)concentration (g/L)	ABE productivity (g/L/h)	ABE (butanol)yield (g/g)	
*SACFH (54.3 g/L sugars)	−	−/−	(1.4)	—	—	30
SACFH + overliming (46.3 g/L sugars)	XAD-4 resin	+/−	6.4 (9.3)	0.10	0.21	30
SAEH (30 g/L sugars)	—	−/−	0.7 (3.5)	0.15	0.12	this study
SAEH (30 g/L sugars)	Optipore L-493	+/−	5.5 (14.4)	0.60	0.49	this study
SAEH +45 g/L glucose	Optipore L-493	+/+	16.4 (33.0)	0.69	0.41	this study
8% glucose	Optipore SD-2	−/+	19.5	N/A	(0.24)	21
Control mixed sugar	—	−/−	13.5 (21.5)	0.89	0.33	this study

^*^SACFH, dilute sulfuric acid pretreated corn fiber hydrolysate followed by enzymatic hydrolysis.

+, positive; −, negative or not available.
